# Epidemiological and therapeutic studies on sheep lice in Sayint district, South Wollo Zone, Northeast Ethiopia

**DOI:** 10.3389/fvets.2022.1008455

**Published:** 2022-11-03

**Authors:** Simegn Legesse, Mussie Hailemelekot, Habtamu Tamrat, Yeshwas F. Alemu

**Affiliations:** ^1^School of Veterinary Medicine, Woldia University, Woldia, Ethiopia; ^2^School of Animal Science and Veterinary Medicine, Bahir Dar University, Bahir Dar, Ethiopia

**Keywords:** diazinon, epidemiological and therapeutic studies, sheep lice, ivermectin, Sayint district

## Abstract

An epidemiological (cross-sectional) and therapeutic (randomized controlled field trial) study was conducted on sheep lice in Sayint district, South Wollo, Northeast Ethiopia. The aim of the study was to (i) quantify the magnitude of sheep lice burden and the prevailing lice species, (ii) identify and quantify risk factors affecting lice infestation in sheep, and (iii) evaluate the efficacy of commonly used acaricides (diazinon and ivermectin) against sheep lice infestation. A total of 232 randomly selected sheep, 15 naturally infested sheep, and 80 viable *Bovicola ovis* lice were used for epidemiological, *in vivo*, and *in vitro* based therapeutic studies, respectively. Three naturally infested treatment groups each with five sheep (Group I—treated with diazinon, Group II—treated with ivermectin, and Group III—untreated/control) were used for *in vivo* therapeutic study. Lice count for the corresponding treatment groups was conducted on weekly basis using clinical and parasitological examinations. We used logistic regression to quantify the association between different putative risk factors and lice infestation, and the independent *t*-test and one-way ANOVA to compare the within and between treatment group mean lice count variations. The overall prevalence of sheep lice in the study area was 48.3%, where *Bovicola ovis* (83%) was the dominant lice species. Hair length, body condition, agroecology, and season were significantly (*P* < 0.05) associated with sheep lice infestation. Analysis of variance revealed that mean lice count significantly (*P* < 0.05) varies between treatment groups. A significant (*P* < 0.05) low mean lice cunt was recorded in diazinon- and ivermectin-treated groups when compared to untreated group. The *in vivo* efficacy of ivermectin (81%) was lower than diazinon (99%) when compared to the efficacy standard limit (98–100%). However, no significant mean lice count variation was recorded between the two groups. *In vivo* (99%) and *in vitro* (95%) assay evidence revealed that diazinon was highly effective for the treatment of sheep lice. According to this study, it can be concluded that the magnitude of sheep lice burden in Sayint district was found to be high and this could have a potential negative impact on sheep productivity and health performances. Thus, applying an appropriate intervention measure including the right choice of effective acaricides could help to control sheep lice in the study area.

## Introduction

Small ruminants in Ethiopia constitute about 30% of the total livestock population of the country and provide 46% of the value of national meat production, 14% of milk consumption, and 58% of the value of hide and skin production (1, 2). Due to their high fertility, short generation interval, and adaptation even in harsh environment, sheep have a crucial role as insurance for income generation to purchase food during season of crop failure and to meet seasonal purchase, such as improved seed, fertilizer, and medicine for rural household (3).

Even though sheep are the most important components of Ethiopian farming system, their production and productivity are constrained by interlinked technical, institutional, and socioeconomic factors. Parasitic diseases of sheep are the major technical constraints affecting sheep production and productivity. In this regard, ectoparasites of sheep (lice, mange mites, and ked) are common health problems widely distributed in all agro-ecological zones in Ethiopia (4). They are considered as a potential animal health threat and pose a serious economic problem in sheep production and the tanning industry through causing morbidity and mortality, decreased production and productivity, downgrading and rejection of skins (5, 6). *Bovicola ovis* is one of the most common lice found on sheep. They feed by chewing on the skin surface and surface debris and produce itching, irritation, and possible hair loss. An allergic skin hypersensitivity reaction to lice is another cause for “Cockle” in processed sheep skins (4). Cockle is the commonly reported disease in Ethiopia (7, 8).

Control of sheep ectoparasites is currently an integration of sheep husbandry, farm management, and insecticide use (9). Chemical control remains the most important and widely used strategy against most ectoparasites around the world, including Ethiopia. However, studies have shown that multiple resistance mechanisms in ectoparasites confer resistance to a range of insecticide classes (9). For instance, the widespread use of chemicals can lead to the sequential development of resistance to *Bovicola ovis* (10). Usually, resistance will be suspected through lack of efficacy during clinical use. However, lack of efficacy could also occur due to inadequate application of a product which leads to resistant ectoparasite species against acaricides (11).

Sayint district is one of the districts in South Wollo Zone known for its potential in sheep production. Mixed crop-livestock is the dominant production system in the area, where indigenous sheep breeds are the major livestock types kept under free grazing, low input, and output husbandry practices (12). In response to the high prevalence of ectoparasites in Ethiopia, the Ministry of Agriculture and Rural Development had designed and launched an ectoparasite control program in Amhara, Tigray, and Afar region in 2005 and in Oromia in 2010 (13). Despite such national and regional efforts, some reports (3, 14) showed that ectoparasites still remain the major challenges affecting small ruminant production in Amhara region in general and Sayint district in particular. Besides, chemical treatment using diazinon and ivermectin is widely used against sheep ectoparasites in Sayint district and other parts of Ethiopia. Nevertheless, farmers in the study area mentioned that these acaricides were not effective in controlling sheep lice infestation.

Therefore, based on the above background, this study was initiated to meet the following objectives: (i) to quantify the magnitude of sheep lice infestation and identify the prevailing lice species, (ii) identify and quantify the associated risk factors affecting lice infestation in sheep, and (iii) evaluate the efficacy of two commonly used acaricides (diazinon and ivermectin) in the control of sheep lice infestation.

## Materials and methods

### Study area

**Figure 1:**
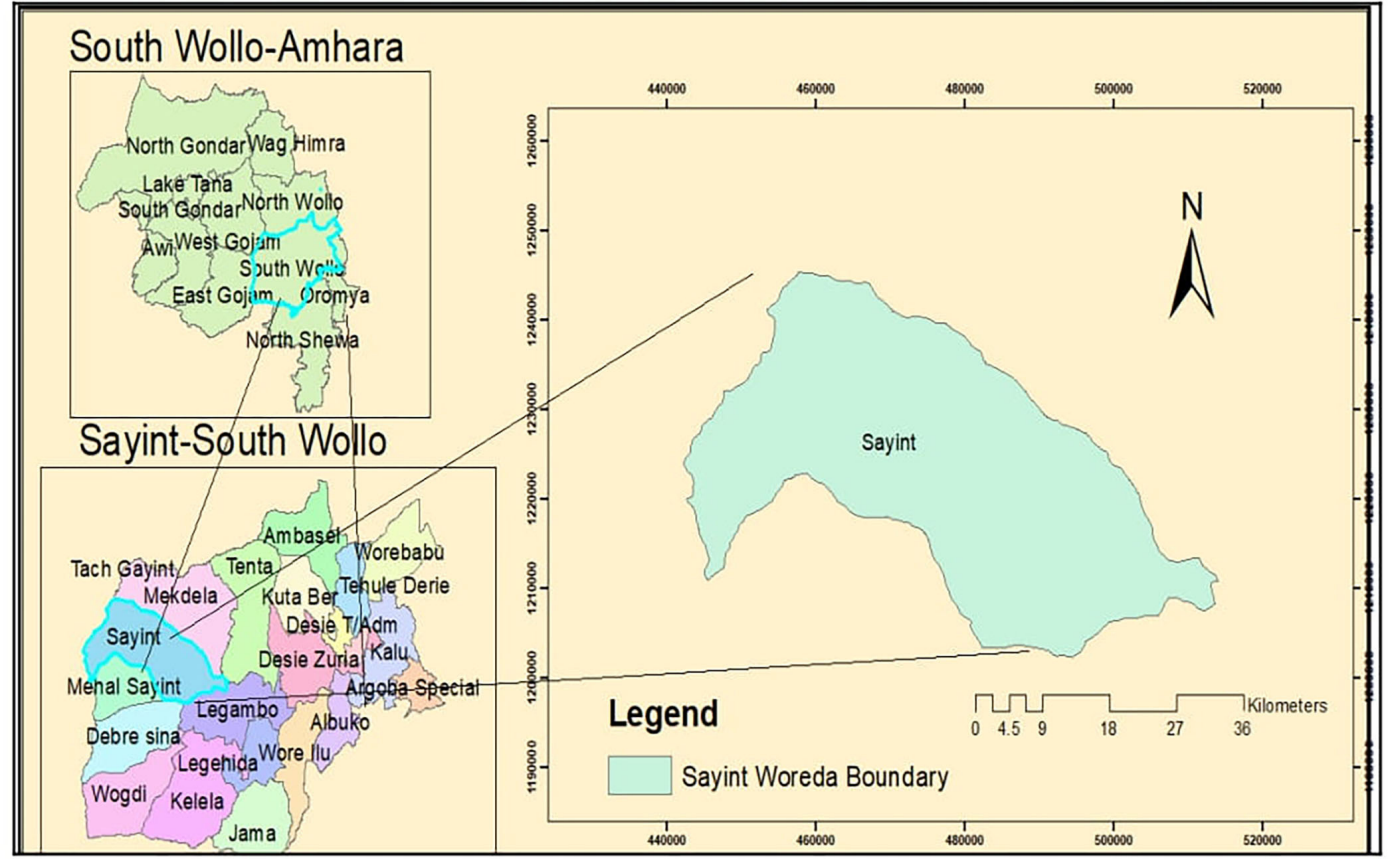
Location map of the study area.

The study was conducted in Sayint district which is found in the western part of south Wollo zone, Amhara National Regional State, which is located 590 km away from Addis Ababa, the capital city of Ethiopia (Figure 1). The altitude of the district ranges from 500 to 4,247 meter above sea level (m.a.s.l). The area constitutes three agroclimatic zones, highland (>2,500 m.a.s.l), midland (1,500–2,500 m.a.s.l), and lowland (<1,500 m.a.s.l), which accounts 42.8, 34.6, and 22.6% of the total area coverage, respectively (15). The average temperature of the district ranges from 4 to 40°C, and annual rainfall ranges from 800 to 1,000 mm (16). The main rainy season in this area is between early June and the end of September, with small rain distribution between early February and end of April (15).

### Study animals

Local Wollo sheep breed and their crosses with Awassi breed of both sexes and all ages managed under extensive production system found in Sayint district were the study animals. South Wollo is known for its sheep production potential sharing 18.4% of Amhara region's total sheep population with 1.86 million sheep (17). In the study area, sheep are mainly reared for income generation from the sale of lambs at market age, although they are important as a source of food, manure, and socio-cultural benefits (18). Crossbreeding of local Wollo sheep with Awassi breed is being promoted in the study areas to improve the livelihood and economy of small holder farmers for wool production (19). According to Gizaw et al. (20), Wollo sheep is among the 14 sheep types in Ethiopia, classified under sub-alpine short fat-tailed group.

### Epidemiological study

#### Study design and sampling strategy

A cross-sectional study design was carried out between October 2018 and April 2019 to quantify the prevalence of sheep lice and identify the major lice species and the associated risk factors affecting sheep lice infestation in the study area. Sex, age, hair length, body condition, housing management (separate or mixed), agroecology (highland, midland, and lowland), and season of sampling (wet and dry season) were recorded and documented along with the sample collection format. Sheep age was classified as young (<1 year) and adult (> 1 year) based on their dentition according to Gatenby (21). Likewise, body condition scores were estimated as poor or good according to Russel (22).

For this study, Sayint district was selected purposively based on its sheep production potential. List of administrative kebeles and villages, and sheep producer households were taken from Sayint district agricultural office. Kebeles and villages are the fourth- and fifth-level administrative units of Ethiopia, respectively. Three-stage (kebele, village, and households) stratified sampling technique was applied based on three agroecologies. Accordingly, 34 *kebeles* were stratified based on agroecology (nine from highland, nine from midland, and sixteen from lowland).

Three *kebeles, one kebele* from each agroecology, were selected randomly, namely “*Guameda”* (*low land*), “*Yegoda”* (*midland*), and *Feresbar* (*high land)* each of which constitutes four, three, and three villages, respectively. One village from each *kebele* (*Yegodo, Yegoda*, and *Ada*) was randomly selected. Sheep were randomly selected from their flock. During the study period, the selected *villages* were visited and sampled twice in October 2018 and January 2019 representing the wet and dry seasons, respectively.

#### Sample size estimation

To study the epidemiology of sheep lice in Sayint district, the required sample size was estimated according to Thrusfield (23), as follows:


$$\begin{array}{l} n=\frac{\left({\left(1.96\right)}^{2}\left[Pexp\left(1-Pexp\right)\right]\right)}{d^{2}} \end{array}$$

where Pexp = expected prevalence, *d* = absolute precision, and *n* = sample size, taking an expected prevalence of 6.94% in Wondmnew et al. (24), confidence level of 95%, 5% desired precision level, and significance level (*p* < 0.05). The required sample size was 99 sheep. However, to improve the precision of our estimate, a total of 232 sheep were used for this study. Samples were proportionally drawn from the three *kebeles*, which included 100, 67, and 65 sheep from Gumeda, Yegoda, and Feres Bet, respectively.

### Therapeutic study

#### Description of treatments and experimental units

After conducting the epidemiological survey and observational assessments, this therapeutic study was designed according to Abu et al. (25) and Holdsworth et al. (26). An *in vivo* controlled field and *in vitro* lab trial were conducted on naturally infested sheep and viable lice, respectively. Accordingly, the efficacy of diazinon and ivermectin was evaluated against sheep lice using *in vivo* and *in vitro* trials. These two candidate acaricides were sourced from local veterinary vendors, which were widely available and commonly used for the treatment of ectoparasites in the study areas. The test acaricide, vetazinon (60% diazinon) contains an active ingredient of 600 g/l diazinon, was produced from Adamitulu pesticides processing share company, Ethiopia. It was manufactured in 19 July 2018 with batch number A-2953-526 and had 2 years of shelf life from date of manufacture. Avomectin (ivermectin 1%) was manufactured by Bash Pharmaceutical Co. Ltd, Khartoum, Sudan, Soba industrial Zone on 03, 2018, batch number 18007013 and had 2 years of shelf life. It was imported by Rang vet Plc, Addis Ababa, Ethiopia.

#### *In vivo* evaluation of diazinon and ivermectin

##### Experimental design and treatment formulation

A completely randomized design (CRD) was used to evaluate the efficacy of diazinon and ivermectin against sheep lice. Fifteen naturally infested local Wollo sheep breed were purposively selected and randomly allocated into three treatment groups (five sheep for each) by a lottery method. Accordingly, the first group was sprayed with diazinon 60% using knapsack sprayer, the second group was given 1% ivermectin injection at a dose rate of 0.2 mg kg^−1^ of body weight subcutaneously as per manufacture's recommended dose, and the third group was left untreated as control. Lice count was performed every 7 days for both treatment and control groups. It was recorded starting from day 0 prior to treatment, days 7, 14, 21, and 28 according to Feyera et al. (27) and Adugna et al. (28).

##### Inclusion criteria and management of experimental animals

Male local sheep naturally infested with lice ≥100, aged between 2 and 4 years, having moderate body condition with short-to-medium hair size and not recently treated with any acaricide, were selected for the experimental study (25, 27). During therapeutic trial, all experimental animals were identified with ear tags and all groups were kept under farmer's management system.

##### Lice burden estimation

Lice burden estimation was conducted by dividing and marking the body of the experimental animal into five parts: neck, shoulder, whither, flank, and ramp after marking four partings per site on both sides of the body (25). Experimental animals were visually examined for lice infestation, and lice from individual animal was counted on the body parts with naked eyes and recorded separately for each treatment groups. Overall, twenty sites on each side of the body were examined by parting the fleece about 10 cm and counting all live lices observed. The total count from 40 sites constitutes the body count for each animal. The total lice count per animal was estimated by summation of the lice number at each site (26).

##### Drug efficacy evaluation

The efficacy of diazinon and ivermectin was evaluated on the basis of clinical and parasitological evidence (29, 30) on day zero (day of treatment) and on days 7, 14, 21, and 28 post-treatments. Lice count for the above corresponding days was conducted according to Holdsworth et al. (31). Finally, lousicidal activity was checked using arithmetic mean louse count for diazinon and ivermectin group separately along with control group which was calculated according to CVMP (32) as follows:


$$\begin{array}{l} \text{\%Efficacy}={\frac{\left(\text{C}-\text{T}\right)}{\text{C}}}^{*}100 \end{array}$$

where C = mean number of lice surviving in the control group and T = mean number of lice surviving in the treated group. Thus, mortality ranging from 98 to 100% indicates susceptibility and mortality of <98% is suggestive of the existence of resistance, and further investigation is needed (33).

#### *In vitro* evaluation of diazinon

##### Lice collection and treatment formulation

The *in vitro* evaluation of diazinon was conducted as per the protocol described by Levot (10). Sufficient live and motile lice were collected manually from naturally infested sheep and immediately taken into the laboratory for species identification and *in vitro* therapeutic evaluation. Accordingly, 80 *Bovicola ovis* lice were randomly allocated into treatment and control groups, each group contains four replicates having 10 lice placed on each petri dish (10 lice/dish). Then, diazinon was diluted in water according to the manufacturer's recommendation (1:1,000), and lice in the treatment group were immersed completely in 0.5 ml of this solution of 60% diazinon for 1 min (25, 34), whereas lice in the control group were kept in the same amount of distilled water. After 1 min, the solution was soaked and dried using Whatman filter paper. Then, the vital signs in which lice exhibited after treatment were checked at 10, 30, 60, and 120 min, including at 6 and 12 h of contact time under microscope (34).

##### Recording of vital signs

Lice vital sign recording and mortality in each visiting time were conducted using a highly stringent criteria (35). For this study, active lice were defined as if no changes in their levels of activity or behavior were observed post-treatment, whereas death of a louse was defined as the complete absence of any vital signs, such as gut movement or movement of antennae or legs (category 4), with or without stimulation using forceps (category 3) after 10, 30, 60, and 120 min as well as after 6 and 12 h of contact time with diazinon. Finally, the percentage mortality was calculated using a formula described by Islam et al. (36) and Askale (37), as follows:


$$\begin{array}{l} \text{\% Mortality}=\frac{\text{Number of dead lice}}{\text{Total number of lice}}\times 100 \end{array}$$

where the insecticidal effect of diazinon was classified as “strong” when mortality is >80%, “moderate” when mortality 80–60%, “weak” when mortality 60–40%, and “little or no activity” when mortality <40%.

### Data collection

#### Clinical examination

Data were collected in two seasons (October and January) to observe the seasonal lice infestation variation between the late rainy and dry seasons of the area. After proper restraining, clinical examination of each sheep was done using multiple fleece partings in the opposite direction in which sheep's hair or wool normally rests. The skin was inspected carefully with naked eye and palpated across all parts of the sheep for the presence of ectoparasites and gross lesions that could be suggestive for clinical form of parasitic infestation (38). Sheep having the parasite or lesions like alopecia and itching was considered as positive.

#### Parasitological examination

Careful examination of the animal body, especially neck, shoulder, breast, ribs, back, and flank, and rump areas was performed to detect the presence of lice by parting the body hair. Lice were collected manually and added into labeled universal bottles with 70% ethanol. Collected samples were dispatched to Bahir Dar Animal Health Diagnostics and Investigation Laboratory for parasitological examination. Lice identification was done using stereo microscope according to the protocol described by Wall and Shearer (39).

### Data management and analysis

Raw data obtained during sample collection and laboratory identification were checked, coded, and entered into Microsoft Excel spreadsheet and then exported to STATA version 15 for analysis. For epidemiological study, we used logistic regression to examine and quantify the association between lice infestation and the associated risk factors, whereas for therapeutic study, independent *t*-test and one-way ANOVA were employed to evaluate mean lice count variations among treatment groups.

First, independent variables were screened using univariable logistic regression at *p* < 0.25 according to Zhang (40). Those screened variables by univariable logistic regression were further evaluated by multivariable logistic regression at *p* < 0.05 to examine their independent effect after the effect of other variables was controlled.

The final model was achieved through stepwise backward elimination of insignificant variables (*p* ≥ 0.05) for each outcome variable. Confidence intervals for the corresponding odds ratios (ORs) were calculated using Wald statistics (41). Before establishing the final model, multicollinearity among predictor variables was checked using variance inflation factor (VIF). Values of VIF exceeding 2.5 are often regarded as indicating multicollinearity in logistic regression (42). Moreover, the goodness-of-fit test for the logistic regression model was examined using the Hosmer and Lemeshow test (43).

## Results

### Epidemiological study

#### Prevalence and species composition of sheep lice

A total of 232 sheep were examined to quantify the prevalence and species composition of sheep lice and to identify its associated risk factors. The overall prevalence and species composition of sheep lice in the study area are summarized in Tables 1, 2. The overall prevalence of sheep lice in *Sayint* district was 48.3% (112/232), in which relatively the highest (66.2%) prevalence was recorded in Feres Bet *kebele* (Table 1). *Bovicola ovis* was the dominant (83%) lice species found in the study area followed by mixed infestation *of Bovicola ovis* with *Linognatus ovillus* (17%). No sole infestation with *Linognatus ovillus was* found by this study (Table 2).

**Table 1:** Prevalence of sheep lice across sampling locations (*kebeles*) in Sayint district, South Wollo, Ethiopia.

**Sampling location (*Kebeles*)**	**Number of sheep sampled**	**Positive**	**Prevalence (%)**
Feres Bet	65	43	66.2
Yegoda	67	40	59.7
Guameda	100	29	29
Total	232	112	48.3

**Table 2:** Species composition of lice species in Sayint district, South Wollo, Ethiopia.

**Lice species identified**	**Number positive (*n*)**	**Proportion (%)**
*Bovicola ovis*	93	83.04
Mixed infestation (*Bovicola ovis + Linignatus ovillus)*	19	16.96
Total	112	100
Negative	100	–

#### Factors affecting sheep lice infestation in sheep

Six potential risk factors were significantly (*P* < 0.25) associated with sheep lice infestation using univariate logistic regression. These included hair length, housing management, body condition, agroecology, and season (Table 3).

**Table 3:** Univariable logistic regression analyses of risk factors associated with sheep lice infestation in Sayint district, South Wollo, Ethiopia.

**Variables**	**Categories**	**Odds ratio**	**95% CI**	***P*-value**
Age	Adult	Ref		
	Young	1.4	0.8–2.4	0.17
Hair length	Short	Ref.		
	Medium	2.3	1.4–4.1	<0.001
Housing management	Separate	0.4	0.2–0.8	<0.001
	Mixed	Ref.		
Body condition	Poor	2.5	1.4–4.3	<0.001
	Good	Ref		
Agro ecology	Highland	4.0	2.1–7.6	<0.001
	Midland	5.0	2.5–10	<0.001
	Lowland	Ref		
Season	Wet	1.8	1.0–3.0	0.02
	Dry	Ref		

However, when those significant risk factors at univariable logistic regression were further evaluated by multivariable logistic regression, only hair length, body condition, agroecology, and season were significantly (*p* < 0.05) associated with lice infestation (Table 4). Lice infestation significantly differed with hair length: High infestation (OR = 2.2, 95% CI: 1.2–3.9, *p* < 0.001) was recorded in sheep with medium hair size. Sheep lice infestation was significantly higher in midland (OR = 4.4; 95% CI: 2.1–9.1; *p* < 0.001) and highland (OR = 3.4; 95% CI: 1.7–6.7; *p* < 0.001) agroecology, when compared to the lowland agroecology (Table 4).

**Table 4:** Multivariable logistic regression analyses of risk factors associated with sheep lice infestation in Sayint district, South Wollo, Ethiopia.

**Variables**	**Categories**	**Odds ratio**	**95% CI**	***P*-value**
Hair length	Short	Ref.		
	Medium	2.2	1.2–3.9	<0.001
Body condition	Poor	1.9	1.1–3.5	0.02
	Good			
Agroecology	Lowland	Ref.		
	Midland	4.4	2.1–9.1	<0.001
	Highland	3.4	1.7–6.7	<0.001
Season	Dry	Ref		
	Wet	2.0	1.1–3.6	0.01

### Therapeutic study

#### *In vivo* efficacy of diazinon

**Figure 2:**
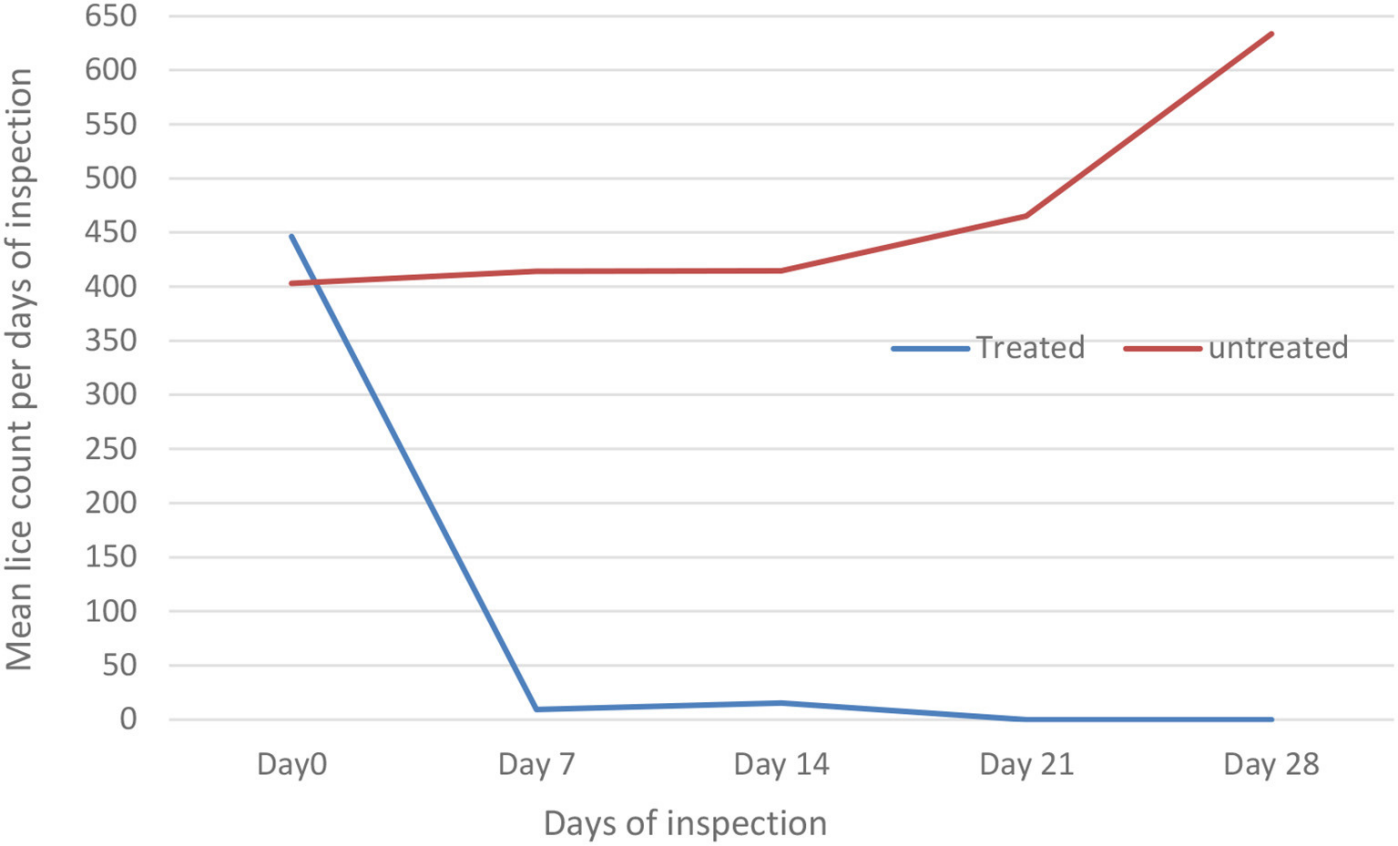
Effect of diazinon treatment on sheep lice infestation (mean lice count) among treated and untreated groups per days of inspection in Sayint district.

The effect of diazinon treatment on lice infestation was evaluated using the independent *t*-test compared with the untreated group (Table 5; Figure 2). The overall *in vivo* efficacy of diazinon was 99%. Before treatment, there was nearly similar mean lice burden on treated and untreated groups. However, except 14 days post-treatment, there was a significant (*p* < 0.05) reduction in mean lice burden on treated sheep with diazinon when compared to the untreated (control) group, whereas mean number of lice burden on untreated sheep was progressively increasing until the end of the experiment (Table 5).

**Table 5:** Effect of diazinon treatment on sheep lice infestation, mean lice count among treated and untreated group in Sayint district, South Wollo, Ethiopia.

**Mean lice count** ±**SD**					
**Treatment days**	**Treated**, ***N*** = **5**	**Untreated**, ***N*** = **5**	***t***	**Df**	***P*** **-value**
Day 0	446 ± 283	403 ± 163	0.29	6	0.086
Day 7	9 ± 3	414 ± 317	−2.85	4	<0.001
Day 14	15 ± 10	415 ± 131	−6.80	4	0.055
Day 21	0.00 ± 0.00	465 ± 178	−5.83	4	0.002
Day 28	0.00 ± 0.00	634 ± 224	-−6.31	4	0.001

SD, standard deviation; df, degree of freedom.

#### *In vivo* efficacy of ivermectin

**Figure 3:**
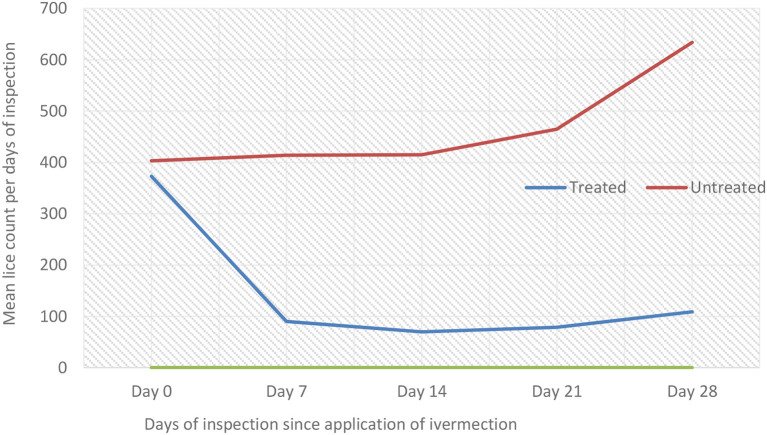
Effect of ivermectin treatment on sheep lice infestation (mean lice count) among treated and untreated groups per days of inspection in Sayint district.

The therapeutic effect of ivermectin on lice infestation was evaluated using the independent *t*-test as compared to untreated groups (Table 6; Figure 3). The overall *in vivo* efficacy of ivermectin was 81%. Before treatment, there was nearly similar mean lice burden on treated and untreated groups. A dramatic reduction in mean number of lice was observed on day 7 post-ivermectin injection; however, it started to increase on days 14, 21, and 28 (Table 6). Mean lice count reduction was significant (*P* < 0.05) at days 7, 21, and 28 between treated and untreated groups (Figure 3; Table 6).

**Table 6:** Effect of ivermectin treatment on sheep lice infestation, mean lice count among treated and untreated group in Sayint district, South Wollo, Ethiopia.

**Mean lice count** ±**SD**					
**Treatment days**	**Treated**, ***N*** = **5**	**Untreated**, ***N*** = **5**	***t***	**df**	***P*** **-value**
Day 0	373 ± 33	403 ± 163	−0.409	4.335	0.111
Day 7	90 ± 39	414 ± 317	−2.275	4.120	<0.001
Day 14	70 ± 13	415 ± 131	−5.860	4.078	0.056
Day 21	79 ± 8	465 ± 178	−4.837	4.015	0.003
Day 28	108 ± 15	634 ± 224	−5.228	4.036	0.001

SD, standard deviation; df, degree of freedom.

#### Analysis of inter- and intra-treatment group mean lice count variations

**Figure 4:**
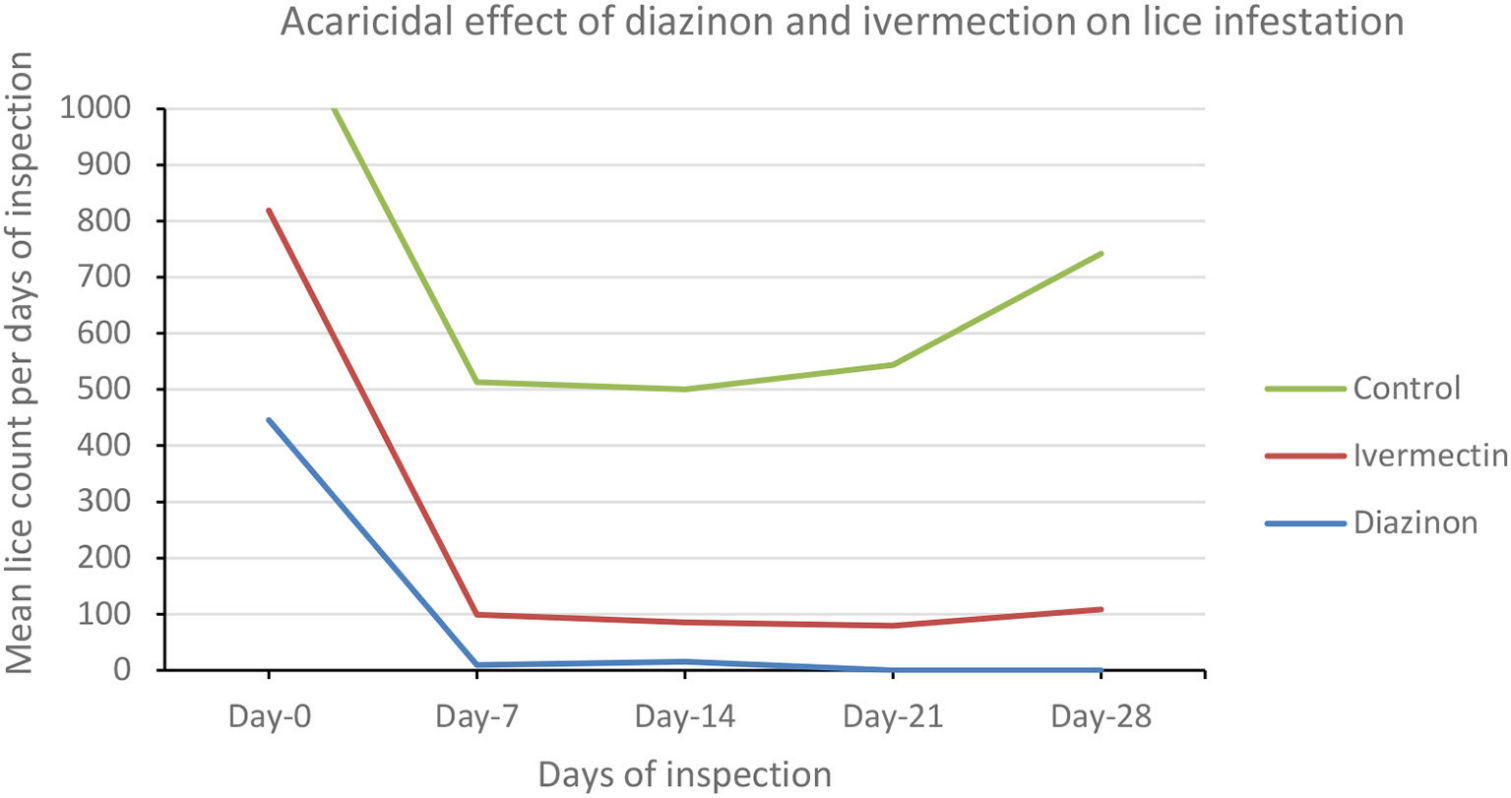
Line graph showing the trends of lice infestation (mean lice count) across treatment groups.

Comparative mean lice count among the three treatment groups was evaluated using one-way ANOVA (Table 7; Figure 4). There were a significant (*P* < 0.05) mean lice count variations between treatment groups (Table 7). However, the Bonferroni test showed that the overall mean lice count reduction was not significant (*P* > 0.05) between diazinon- and ivermectin-treated groups (Table 7). Overall, treatment with diazinon and ivermectin was highly effective at the first week (day 7) post-treatment when compared to other observation periods.

**Table 7:** Comparative effect of diazinon and ivermectin treatment on lice infestation in sheep in Sayint district, South Wollo, Ethiopia.

**Treatment group**	**NPG**	**Mean estimated lice count**				
		**LC-Day 0**	**LC-Day 7**	**LC-Day 14**	**LC-Day 21**	**LC-Day 28**
Diazinon	5	446 ± 283^b^	9 ± 3^b^	15 ± 10^b^	0.00 ± 0.00^b^	0.00 ± 0.00^b^
Ivermectin	5	373 ± 33^b^	90 ± 39^b^	70 ± 13^ba^	79 ± 8^b^	108 ± 15^b^
Control (untreated)	5	403 ± 163^b^	414 ± 317^a^	415 ± 131^a^	465 ± 178^a^	634 ± 224^a^

NPG, number of experimental animals per group; LC, lice count; numerical values are mean lice count ± SEM (standard error of mean), and the values with different superscript letters (a and b) within the same column and class are significantly different (*P* < 0.05).

#### *In vitro* efficacy of diazinon

**Figure 5:**
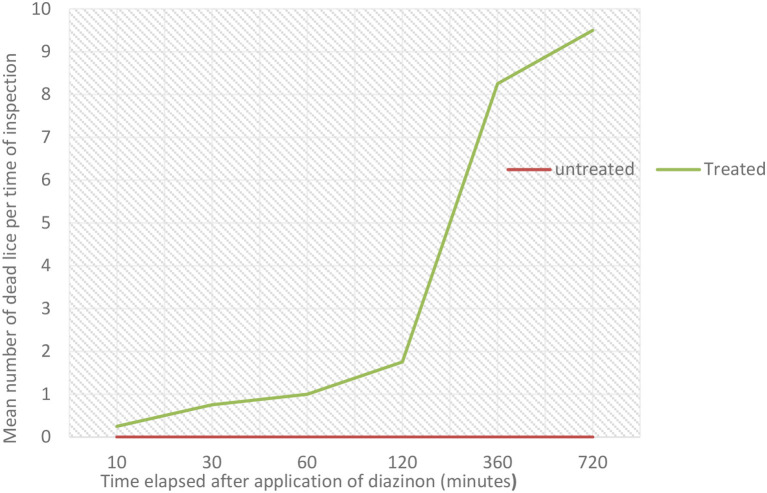
Effect of *in-vitro* diazinon treatment on the mean number of dead lice per time of inspection.

The *in vitro* efficacy of diazinon against sheep lice was evaluated based on the vital signs' lice exhibit during the test. All lice in the control group had survived during the observation period. The study showed that the tested acaricide killed 2.5% of lice at 10 min, 7.5% at 30 min, 10% at 60 min, 17.5% at 120 min, 82.5% at 360 min (6 h), and 95% at 720 min (12 h). There was marginally significant variation in percentage mortality of lice between diazinon treated and untreated group throughout the study periods (*p* = 0.05). The highest mortality was seen within the treatment group at 720 min (12 h). The overall *in vitro* efficacy of diazinon was calculated to be 95%. The *in vitro* post-treatment mean number of dead lice per time period is shown in Figure 5. The mean number of dead lice in the treated group was slightly increased from 30 to 120 min, and high mortality percentage was seen between 120 and 360 min. There was also very high mortality from 360 to 720 min (Figure 5). However, there was no any change in untreated group throughout the study period.

## Discussion

### Epidemiological evidence

In the present study, the overall prevalence of sheep lice in the study area was 48.3% which is found consistent with Bedaso et al. (44) and Assefa (45) in Tiyo and Dikiss of Arsi Zone (49%) and Eticha et al. (46) in Arsi Highland areas (54%). However, it was found higher than the reports of Mohamed Saied (47) in Ginir District of Bale Zone (5.5%), Bereket et al. (48) in Gamo Gofa Zone (4.6%), Fikre et al. (49) in Afar region (11.6%), Tamerat and Zeryehun (50) in Eastern Ethiopia (21.4%), Abebe et al. (1) in Tigray region (1.3%), Tadesse et al. (51) and Desalegn et al. (52) in Kombolcha (28.5%, 21.7%), Amuamuta et al. (53) and Dawit et al. (54) in Bahir Dar (11.5%, 3.8%), Teshome (55) in Gondar (13%), Eshetu et al. (56) in Wogera (12.7%), and Wondmnew et al. (24) in Kutaber (6.9%). However, this report was found lower than Bedada et al. (13) and Fentahun et al. (57), who reported 83% and 57% prevalence of sheep lice in Arsi Zone and Gondar, respectively. The possible reason for such discrepancies in the prevalence of lice across study areas might be attributed to variations in sheep management and healthcare practices of the farmers in the study areas, season and agro-ecological variations among studies, presence or absence of ectoparasite prevention and control programs, presence or absence of adequate veterinary service, and proper use of acaricides.

*Bovicola ovis* (83%) was the dominant lice species identified by the present study, and the rest was mixed infestation of *Bovicola ovis* with *Linognatus ovillus* (17%). A similar finding was also reported by Bekele et al. (58), 83.23% prevalence of *Bovicola ovis* in sheep in Wolmera district and Eticha et al. (46) who reported 86% *Bovicola ovis*, 1.9% *Linognatus ovillus*, and 12% mixed infestation in Arsi, and Bedaso et al. (44) who reported 0.01% *Linognatus ovillus*. However, it is much higher than the reports of Israel et al. (38) and Bedaso et al. (44) who reported 29% and 73% *Bovicola ovis*, respectively.

A significant (*P* < 0.05) association between sheep lice infestation and hair length was found by this study. Those sheep with medium hair were coat more likely to be infested as compared to short ones, which was in lined with other previous report by Bedada et al. (13). According to the present study, poor conditioned sheep were more likely to be infested with sheep lice as compared to well-conditioned counterparts, which agrees with the results of Deferes and Geresu (7), Amare et al. (14), and Fekadu et al. (59), who reported that sheep having poor body condition were more likely to be affected by ectoparasites. Poor body condition can be exacerbated by itching and disturbance during grazing. This condition can compromise the nutritional status of sheep and leads to immunosuppression. However, the present finding contradicts the findings of Bedada et al. (13) who reported that prevalence of *Bovicola ovis* was higher in well-conditioned animals.

In the present study, sheep kept in midland and highland agro-climates were more likely to be infested by sheep lice as compared to lowland agro-climates. This finding was found in line with the reports of Abebe et al. (1), Sertse and Wossene (3), Kumsa et al. (4), and Amare et al. (14), who reported that a higher prevalence of lice was recorded in highland and midland areas.

The reason for higher prevalence in midland and highland might be associated with the temperature and moisture requirement of *Bovicola ovis* as well as shearing practice of farmers in the study area. In midland areas, temperature and moisture are conducive for lice to multiply.

In highland areas, as the climate is very cold, farmers do not practice wool shearing to protect their sheep from cold stress. This practice could predispose sheep to lice infestation.

A significant higher lice infestation rate was observed during dry season as compared to wet season. It was found consistent with the reports of Mohamed Saied (47) and Kassaye and Kebede (60), while it disagrees with the reports of Askale (37) and Bereket et al. (48), who reported that a higher prevalence of sheep lice was recorded in wet season. The significant discrepancy in lice infestation among seasons could be attributed to the availability of animal feeds, where feed scarcity is common in dry seasons. This could expose sheep to poor body condition and immunosuppression which ultimately led to lice infestation.

### Therapeutic evidence

This study evaluated the therapeutic efficacy of diazinon and ivermectin using *in vivo* and *in vitro* assays. The *in vivo* and *in vitro* efficacy of diazinon against sheep lice infestation was 99 and 95%, respectively. The drug significantly reduced lice infestation when compared to untreated group. Hence, considering the statistical and the efficacy standards of Brogdon and Chan (33), the drug was found effective for the treatment of sheep lice (*Bovicola ovis)* and farmers in the study areas can use this drug for the treatment of sheep lice. This result is found consistent with the reports of Aziz et al. (61), who reported that the *in vitro* and *in vivo* efficacy of diazinon was 100%.

The efficacy of ivermectin was 81%, with a significant lice infestation reduction when compared to the untreated group. Although the apparent efficacy of ivermectin was relatively lower as compared to diazinon and the efficacy standard limit (98–100%) recommended by Brogdon and Chan (33), a significant mean lice count reduction was not found between the two drugs. Such discrepancies among statistical and parasitological evidence might require further verification. Nevertheless, the overall *in vivo* efficacy of ivermectin reported by this study was found far lower than the findings of Wondmnew et al. (24), Aziz et al. (61), and Hassan et al. (62), who reported that ivermectin was 100% effective against sheep lice infestation. Besides, lice infestation was gradually increased at 14 days of post-ivermectin injection. A spontaneous increase in lice count after 14 days of treatment could be due to reinfestation, as ivermectin might not kill the egg or nymphal stage of lice which could subsequently developed into adult stage. Most acaricidal preparations are not licensed to kill eggs, and as a result they may hatch and re-establish infestation post-treatment (63).

The reduced efficacy of ivermectin injection against sheep lice could also be associated with indiscriminate and high frequent use ivermectin in the study area. Survey findings revealed that limited drugs were available in local veterinary clinics, where local farmers indiscriminately used ivermectin for the treatment of ectoparasites in sheep and cattle. The importance of these presumed factors associated with drug resistance was stated by Byaruhanga and Okwee-Acai (64). It is also evident that lack of insecticide class rotation and high treatment frequency increases resistance of parasites (64, 65).

### Limitation of the study

Due to logistical limitations, treatment groups were not replicated more than once as a result of limited experimental animals (sheep). The efficacy of ivermectin was evaluated only using *in vivo* trial. Such limitations need to be considered during result interpretation.

## Conclusion

The present study revealed that there was a higher prevalence of sheep lice infestation in the study area, where *Bovicola ovis* was the most prevailing lice species affecting sheep. Hair length, body condition, agroecology, and season were significantly associated with lice infestation in sheep. The *in vivo* and *in vitro* trials showed that diazinon was more effective to treat *Bovicola ovis*, whereas the efficacy of ivermectin was relatively low. Hence, subsequent application of tailor-made intervention toward the identified risk factors with rational choice of effective acaricides could reduce the prevalence of sheep lice infestation in the study area. Further therapeutic research is needed to evaluate the efficacy of ivermectin in the control of sheep lice.

## Data availability statement

The original contributions presented in the study are included in the article/supplementary material, further inquiries can be directed to the corresponding author/s.

## Ethics statement

The animal study was reviewed and approved by College of Agriculture and Environmental Science, School of Animal Science and Veterinary Medicine, Bahir Dar University. Ethical clearance was obtained from Bahir Dar University's Research Ethics Review Committee. Written informed consent was obtained from the owners for the participation of their animals in this study.

## Author contributions

SL conceived the study, collected and analyzed the data, and drafted the manuscript. MH designed the study and revised the manuscript. HT analyzed the data and revised the final manuscript. YA conceived the study, analyzed and interpreted the data, and revised the final manuscript. All authors contributed to the article and approved the submitted version.

## Funding

This work was financially supported by Woldia University.

## Conflict of interest

The authors declare that the research was conducted in the absence of any commercial or financial relationships that could be construed as a potential conflict of interest.

## Publisher's note

All claims expressed in this article are solely those of the authors and do not necessarily represent those of their affiliated organizations, or those of the publisher, the editors and the reviewers. Any product that may be evaluated in this article, or claim that may be made by its manufacturer, is not guaranteed or endorsed by the publisher.
